# Blood identified and quantified in formalin fixed paraffin embedded lung sections using eosin fluorescence

**DOI:** 10.1007/s00418-022-02130-z

**Published:** 2022-08-25

**Authors:** Robert J. Francis, Deborah Ferguson, Sarah Kempster, Joanna Hall, Neil Berry, Kirsty MacLellan-Gibson

**Affiliations:** 1 Biological Imaging Group, National Institute for Biological Standards and Control, Blanche Lane, South Mimms, EN6 3QG UK; 2 Division of Infectious Disease Diagnostics, National Institute for Biological Standards and Control, Blanche Lane, South Mimms, EN6 3QG UK

**Keywords:** Eosin, FFPE, H&E, Erythrocytes, Spectral, Section

## Abstract

Eosin Y is a common stain in histology. Although usually used for colourimetric imaging where the dye is used to stain pink/red a range of structures in the tissue, Eosin Y is also a fluorochrome, and has been used in this manner for decades. In this study our aim was to investigate the fluorescence properties of the dye to enable quantification of structures within formalin-fixed paraffin-embedded (FFPE) tissue sections. To do this, FFPE sections of hamster tissue were prepared with haematoxylin and eosin Y dyes. Spectral detection on a confocal laser scanning microscope was used to obtain the fluorescence emission spectra of the eosin Y under blue light. This showed clear spectral differences between the red blood cells and congealed blood, compared to the rest of the section. The spectra were so distinct that it was possible to discern these in fluorescence and multi-photon microscopy. An image analysis algorithm was used to quantify the red blood cells. These analyses could have broad applications in histopathology where differentiation is required, such as the analysis of clotting disorders to haemorrhage or damage from infectious disease.

**Supplementary Information:**

The online version contains supplementary material available at 10.1007/s00418-022-02130-z.

## Introduction

Eosin Y is a dye that is commonly used in histopathology and is routinely used to stain formalin-fixed paraffin-embedded (FFPE) sections. The dye was first discovered by the German chemist Heinrich Caro in 1874 (Kay 2015). Eosin is known to have fluorescent properties with excitation in the blue/green wavelengths with an absorption maximum of 526 nm (Taniguchi and Lindsey 2018), and emission in the green wavelengths of the visible light spectrum that have been exploited since at least the 1960s (Goldstein 1969; Lev and Stoward 1969). The fluorescent properties of eosin are known to change under certain conditions; for example, a shift in wavelength is seen with liver injury (Ali et al. 2017). Other researchers have shown that eosin in combination with DRAQ5, a far-red nucleic acid stain, when imaged using fluorescence gives similar staining to haematoxylin and eosin (H&E) (Elfer et al. 2016). However, Jakubovsky et al. (2002) observed that the fluorescence from red blood cells can hinder evaluation of the spleen in FFPE sections.

Eosin Y bound to erythrocytes is known to produce a colour change towards red in visible light (Sampias and Rolls 2021). This change is subtle enough to make image analysis and quantification difficult, thus requiring specialist image analysis algorithms (Gray et al. 2015). Fluorescence microscopy has advantages over chromatic microscopy as fluorescence microscopy is captured in monochrome with a fixed filter set; consequently, fluorescence micrographs are easier to quantify and replicate between instruments. In addition, spatial resolution can be reduced with chromatic microscopy because it uses a full-colour camera requiring a Bayer pattern of filters in front of the camera chip (Weber and Menko 2005).

Here we describe a new method that exploits the different fluorescent properties of eosin Y when bound to erythrocytes to quantify blood within H&E-stained tissue sections, and show how this can be combined with other specialised techniques to gain further information from the tissue.

## Methods

### Formalin-fixed paraffin embedded (FFPE) histological sections

Hamster tissue, either from healthy animals or those infected with a genetically characterised strain of SARS-CoV-2/Victoria-01, was obtained in accordance with the Animal Scientific Procedures Act and under Home Office Licence, at a designated establishment in the UK. A single dose representing 5 × 10^4^ 50% Tissue Culture Infectious Dose (TCID50) was administered atraumatically via the injection route. Intact lungs were harvested post-mortem and were fixed for 72 h at room temperature in 10% formal saline (Sigma-Aldrich, St. Louis, MO, USA) and embedded in paraffin wax (VWR, Avantor, Radnor, PA, USA) in accordance with standard histological processes. Sections were cut to a thickness of 4 μm on a microtome (Leica Biosystems, Wetzlar, Germany), mounted onto slides and stained using haematoxylin and eosin Y (both Vector Laboratories, Newark, CA, USA) using the manufacturer’s staining protocol. Sections were then coverslipped (no.1 depth).

To check the staining integrity of the sections, full-colour microscopy was conducted on a widefield microscope equipped with a full-colour camera (model Evos XL Core; Thermo Fisher Scientific, Waltham, MA, USA) using 10× and 40× lenses.

### Spectral fluorescence microscopy

Spectral analysis was performed using a confocal laser scanning microscope equipped with a white light laser for visible excitation (Leica TCS SP8 MP OPO microscope; Leica Biosystems), with a 20×/0.7 numerical aperture lens without immersion. The emission was imaged from the stained samples using a stepwise movement of a 10-nm filter at 10-nm steps across the visible light emission from 480 to 690 nm, when excited with a laser at 470 nm, using a hybrid detector with a fixed gain of 300%. Images were taken at Nyquist resolution and then reduced to 512 × 512 pixels for visualisation.

### Widefield microscopy

Widefield microscopy was conducted using fixed filter sets on an Olympus IX81 inverted fluorescence motorised microscope (Olympus Corp., Tokyo, Japan), using a 40×/0.75 NA lens without immersion equipped with a 16-bit monochrome camera (Hamamatsu Orca Flash 4.0; Hamamatsu Photonics K.K., Hamamatsu, Japan). Green emission was captured using a fluorescein isothiocyanate (FITC) filter set (excitation 460 nm, emission 520-540 nm), and red emission was captured using a tetramethylrhodamine (TRITC) filter set (excitation 580 nm, emission 570-610 nm), without modification.

For comparative full-colour microscopy, a white light source (CoolLED pE-100; CoolLED Ltd., Andover, UK) was used to illuminate the sample with emission filters for blue, green or red in front of the monochrome camera. The three images were then integrated into a composite image using NIH ImageJv2 software (Rueden et al. 2017; Schindelin et al. 2015).

### Two-photon microscopy

Two-photon (2-P) microscopy was conducted using a Leica SP8X confocal laser scanning microscope (CLSM) (Leica Biosystems) equipped with Coherent Chameleon laser (Coherent Inc., Santa Clara, CA, USA) using a 25×/0.95 NA water immersion lens. Excitation was set at 1000 nm, and reflective light emission was filtered using 500- to 550-nm (FITC) and 565- to 605-nm (TRITC) filters.

For the second harmonic generation signal a transmitted light detector was used between wavelengths 495 and 505 nm, at the same time as the reflective light detection. As the signal was relatively weak, a frame accumulation of 10 was used to enhance the signal, with line average of 3.

## Results and discussion

**Fig. 1:**
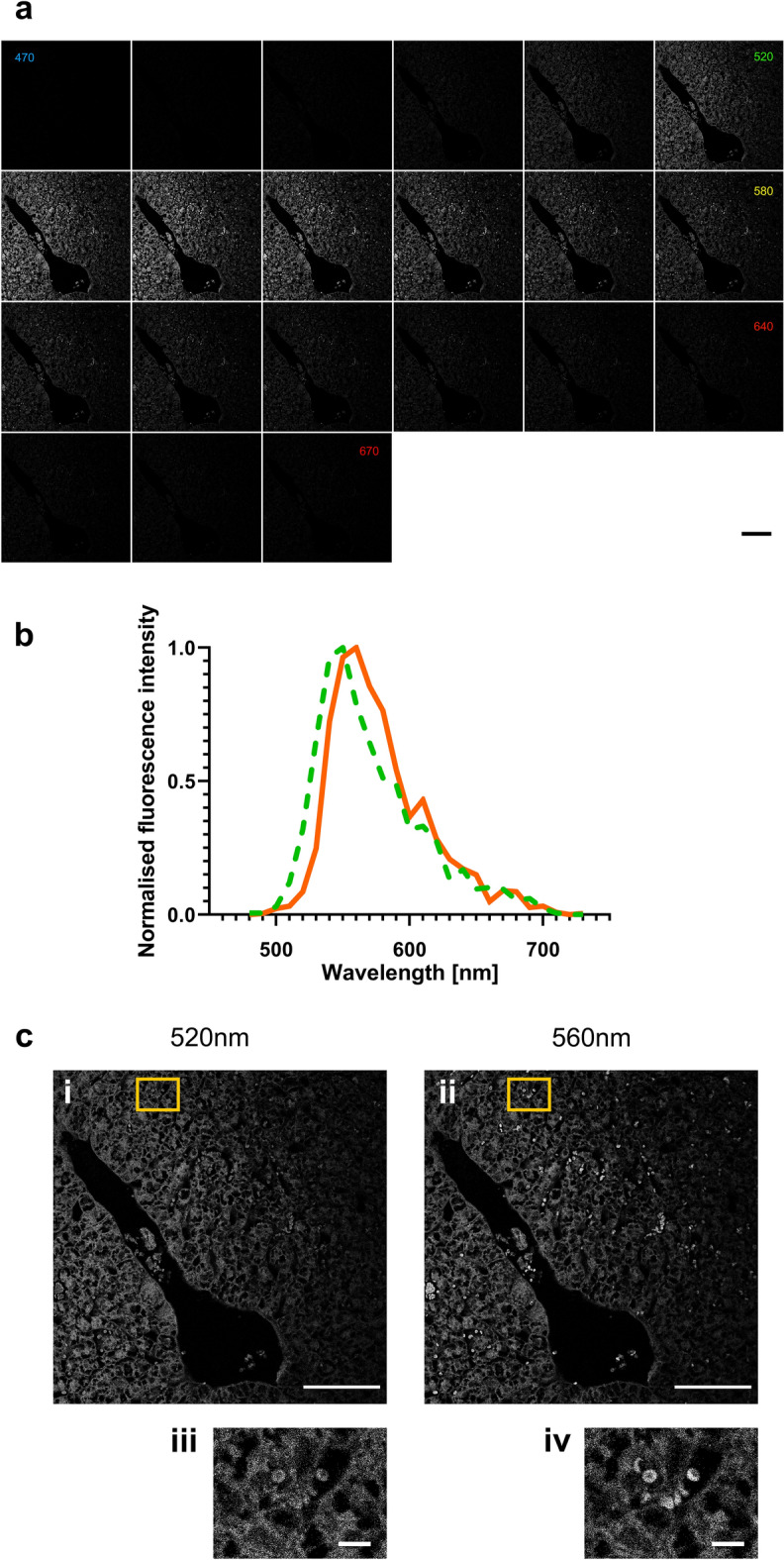
Spectral analysis of eosin. **a** Montage of images of naïve lung at 10-nm intervals with a filtered width of 10 nm across the visible spectrum from 480 to 680 nm, with 470 nm excitation. **b** Example spectra taken from areas of tissue (green dotted line) and from blood (solid orange line), where the red shift is visible in areas where blood is present (seen in the images as brighter with a punctate appearance, with a shift in colour from bright green to a deeper green/yellow). **c** Images taken at 540 nm (**c**, *i*) and 580 nm (**c**, *ii*) scaled by 50% from the original. **c**, *iii* and **c**, *iv* are the original images from the region of interest marked in yellow in image **c**, *i* and **c**, *ii* respectively showing what are presumed to be red blood cells with circular, donut-shaped objects of approximately 5 µm width. Scale bars: 100 µm (**a**, **c**, *i* and **c**, *ii*), 10 μm (**c**, *iii* and **c**, *ii*)

Eosin Y is known to be fluorescent, but its full spectral properties have yet to be extensively explored. To analyse the spectral properties of eosin, we placed a stepwise filter in front of a monochrome detector and moved it across the visible light spectrum (Fig. 1a), with the laser excitation at a fixed point of 470 nm. Spectral analysis with an excitation wavelength of 470 nm was performed on tissue we had previously observed under white light conditions. The white light images revealed tissue with pink staining or deep-red staining where blood was present. We observed that most of the tissue had an emission maximum at 550 nm (Fig. 1b) and appeared green. However, when observing areas where blood was present, there was a red-shift in the fluorescence spectra, resulting in a visual shift towards a deeper green/yellow. Areas where blood was present had shifted to an emission maximum of 570 nm (Fig. 1b). Areas with blood were also found to be brighter, with a punctate appearance (Fig. 1c, ii) compared to the rest of the tissue (Fig. 1c, i), and when magnified, these brighter areas were found to be mainly composed of individual red blood cells (Fig. 1c, iii).

**Fig. 2:**
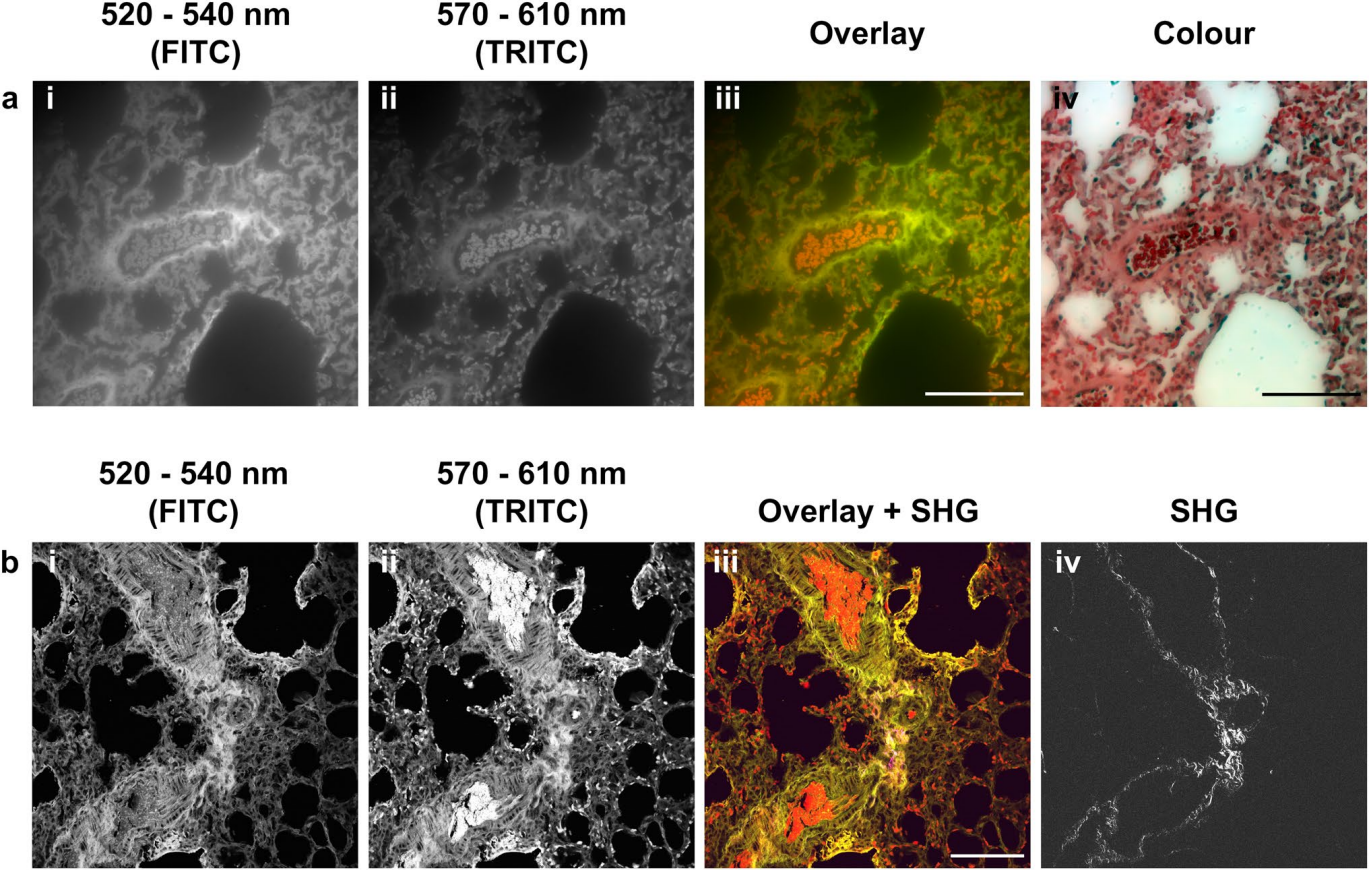
Imaging using fixed filter parameters. **a** Widefield microscopy of the stained sections of naïve lung showing a fluorescein isothiocyanate (*FITC*) signal (**a**,* i*) and tetramethylrhodamine (*TRITC*) signal (**a**, *ii*), and the overlay of the two (**a**,* iii*) with FITC in green and TRITC in red. The full-colour image is shown as a comparison (**a**,* iv*). **b** Two-photon microscopy of the stained sections of diseased lung (day 28 post-SARS-CoV-2 infection) showing once again the FITC signal corresponding to tissue (**b**,* i*) and the TRITC signal corresponding to blood (**b**,* ii*). The blood signal is especially evident in both larger blood vessels and in alveolar capillaries. The second harmonic generation (*SHG*) signal (**b**,* iv*) was also obtained, showing the collagen fibres surrounding the larger blood vessels, within the sections. The overlay (**b**,* iii*) shows all three stains, with FITC as green, TRITC as red and SHG as pink. Scale bar: 100 µm

Once spectral analysis had highlighted the possibility of differentiating blood from the rest of the tissue, we aimed to apply this finding to fluorescent microscopy. Using a standard FITC (520-540 nm) and TRITC (570-610 nm) filter set in widefield fluorescence microscopy (Fig. 2a), we noted that the blood signal was identified specifically by TRITC (Fig. 2a, ii) and that it could be clearly seen when an overlay is produced with a FITC filter (Fig. 2a, iii). The same principles were applied to 2-P microscopy whereby the FITC and TRITC filters were placed in front of the detectors and images produced (Fig. 2b). As 2-P microscopy was used, the capture of the second harmonic generation signal was also achieved (Fig. 2b, iv), which allows for the visualisation of collagen fibres in the tissue section (Campagnola 2011).

**Fig. 3:**
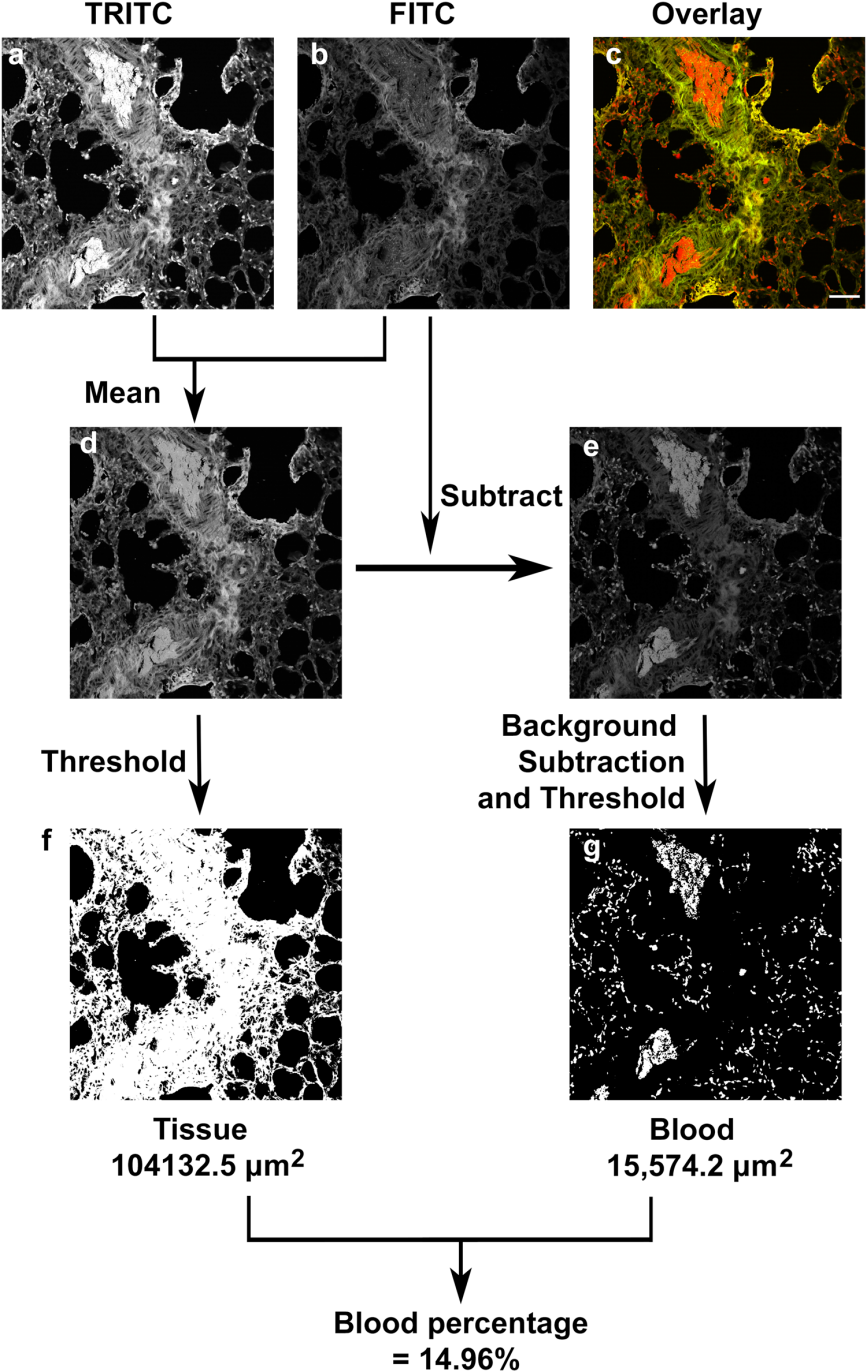
Quantification algorithm for measuring blood within eosin Y stained sections. **a** Is the image from the TRITC channel, while (**b**) is from the FITC channel, with (**c**) the overlay with SHG signal. To quantify the blood signal within the sections, the images are first normalised across grey levels then mean signal of the FITC and the TRITC channels is produced (**d**) and the FITC signal subtracted from the mean signal to produce an image with the specific blood signal above the tissue signal (**e**). To quantify the tissue signal, the mean image (**d**) is thresholded using an automated thresholding method (**f**), in this case Huang algorithm. To quantify the blood signal, the subtracted image (**e**) is again thresholded using an automated thresholding method (**g**), in this case Huang as well. Then the area is recoded in each image, with the percentage of blood to tissue then calculated. The algorithm is provided as supplementary material. Scale = 50 μm and applies to all images

The spectral differences facilitate visualisation of blood clearly within the sections, with the difference in fluorescent properties lending themselves towards quantification. To achieve this, the sample was imaged using the FITC and TRITC filters (Fig. 3a–c), normalised across the grey levels and subsequently averaged together using the mean fluorescence intensity of each (Fig. 3d). The FITC image was then subtracted from the average image, to produce an image where the blood signal is amplified above other signals (Fig. 3e). The average image can then be thresholded using an automated thresholding method, such as the Huang algorithm (Huang and Wang 1995), to quantify the total area of positive pixels in the tissue image (Fig. 3f). Background subtraction and thresholding of the subtracted image (Fig. 3e), in this case using the Huang algorithm, can be used to quantify the pixels that are specific to blood (Fig. 3g). Further examples are shown (Electronic Supplementary Material Fig. 1), and the algorithm is available as electronic supplementary material.

This study is the first to show that the fluorescent properties of eosin Y can be exploited to quantify the intravascular blood within H&E-stained sections of tissue derived from naïve and diseased lungs (day 28 post-SARS-CoV-2 infection) prepared under standard conditions. With the advent of machine learning and automated pathology technologies, simple algorithms that can exploit properties within existing standardised protocols could increase the exploitable data generated from standardised protocols, and promote adoption within digital pathology laboratories.

The importance of being able to quantify blood in tissue has been highlighted in the recent SARS-CoV-2 pandemic where microthrombi and haemorrhage are a distinct pathology (Ackerman et al. 2020; Caramaschi et al. 2021; Dolhnikoff et al. 2020; Klok et al. 2020). Being able to identify and quantify blood in tissue therefore could be a clinical parameter to support clinical diagnostics and enable comparative pathology in animal models.

## Supplementary Information

Below is the link to the electronic supplementary material.

Supplementary file 1 Examples of blood quantification. (**a**–**j**) examples of composite images that have been quantified using the algorithm. (**c**) is presented in Figs. 2b, 3. Scale bar: 50 µm (TIF 58583 KB)

Supplementary file 2 (IJM 3 KB)

## Data Availability

The datasets generated during and/or analysed during the current study are available from the corresponding author on reasonable request.
