# Supplementary figures and images for: Blood identified and quantified in formalin fixed paraffin embedded lung sections using eosin fluorescence
Source: Histochem Cell Biol. 2022 Aug 25;158(4):383–8. doi: 10.1007/s00418-022-02130-z (PMC9406261; doi:10.1007/s00418-022-02130-z)

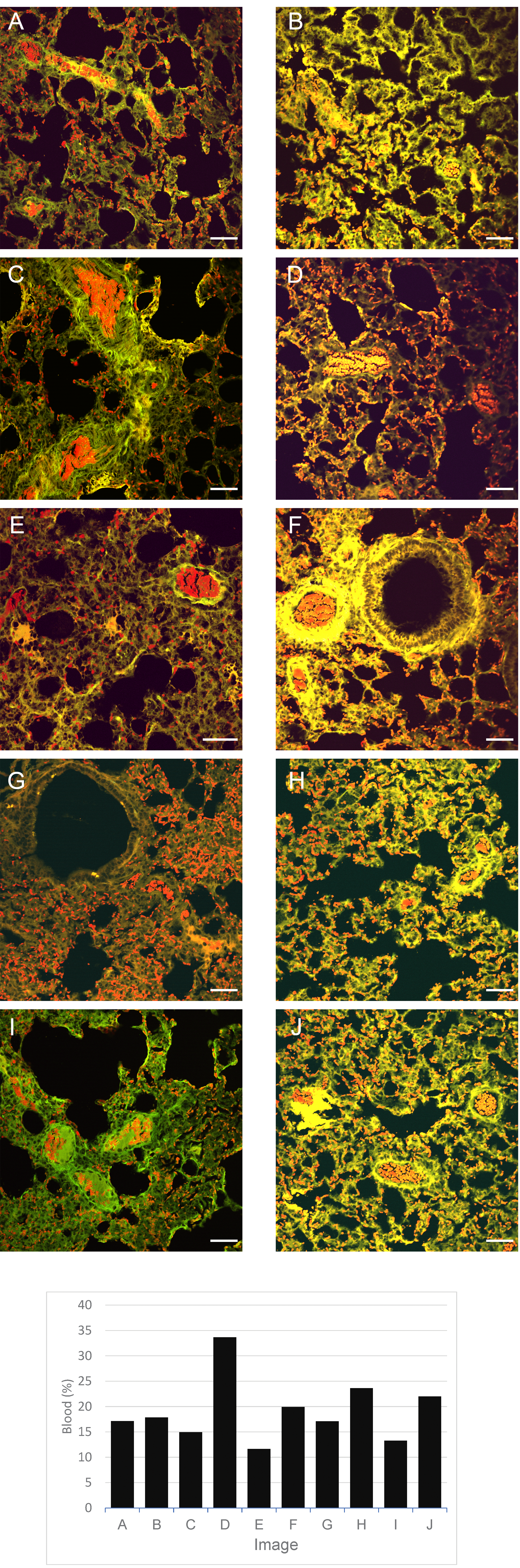

Supplement: Supplementary file 1 — Examples of blood quantification. (a–j) examples of composite images that have been quantified using the algorithm. (c) is presented in Figs. 2b, 3. Scale bar: 50 µm (TIF 58583 KB) [file 418_2022_2130_MOESM1_ESM.tif]
